# Effect of Process Conditions on the Microstructure and Properties of Supercritical Ni-GQDs Plating

**DOI:** 10.3390/ma17184620

**Published:** 2024-09-20

**Authors:** Haoyu Zhong, Cong Fang, Weining Lei, Tianle Xv, Bin He, Linglei Kong, Yiliang He

**Affiliations:** 1 School of Mechanical Engineering, Jiangsu University of Technology, Changzhou 213001, China; hy_zhong_personal@163.com (H.Z.); 18263851593@163.com (C.F.); 18339188030@163.com (T.X.); binhe06@163.com (B.H.); konglingl@nuaa.edu.cn (L.K.); 13485987469@163.com (Y.H.); 2 Jiangsu Key Laboratory of Advanced Material Design and Additive Manufacturing, Jiangsu University of Technology, Changzhou 213001, China

**Keywords:** supercritical CO_2_, electrodeposition technique, GQDs, microstructure, mechanical properties, corrosion resistance

## Abstract

The Ni-GQDs composite plating was created using direct current (DC), single-pulse, and double-pulse power supplies, with GQDs serving as additives under supercritical CO_2_ conditions. A comparative analysis was conducted to evaluate the effects of different electrodeposition power sources on the microstructure and properties of the Ni-GQDs composite plating. High-Resolution Transmission Electron Microscopy (HRTEM) was employed to investigate the distribution of GQDs within the composite plating as well as to analyze d-spacing and diffraction patterns. Scanning Electron Microscopy (SEM) was utilized to illustrate the surface morphology of the plating and assess its surface quality. The grain size and preferred orientation of the plated layer were examined using X-ray Diffraction (XRD), while Atomic Force Microscopy (AFM) was used to evaluate the roughness of the surface. To compare the abrasion resistance of the various plating types, wear amounts and friction coefficients were measured through friction and wear tests. Additionally, corrosion resistance tests were performed to assess the corrosion resistance of each plating variant. The results indicate that the Ni-GQDs-III composite layers produced via double-pulse electrodeposition exhibit superior surface quality, characterized by smaller grain sizes, enhanced surface flatness, reduced surface roughness, and improved resistance to wear and corrosion.

## 1. Introduction

Supercritical fluids display both liquid solubility and gas diffusivity [1,2,3]. They are also able to enter micropores quickly because of their low viscosity and low surface tension [4]. Moreover, variations in temperature, pressure, and polarity have an impact on the solubility of supercritical fluids [5]. The supercritical state of CO_2_ is reached when its temperature surpasses 31.26 °C and its critical pressure increases over 7.39 MPa [6]. Composite electrodeposition technology [7,8,9] refers to the process of simultaneous deposition of nanoparticles and metal ions, whereby the unique properties of the nanoparticles can be added to the metal coating, strengthening the wear resistance, corrosion resistance, and hardness of the metal covering. The combination of composite electrodeposition with supercritical CO_2_ is crucial for enhancing the mechanical, chemical, and surface quality properties of the plating [10,11]. S. Pandiyarajan et al. [12] demonstrated an approach to enhance the dispersion of GO nanodisks in the electrolyte without the use of additives while also accelerating the Ni/GO plating’s deposition using a new US-SC-CO_2_ feed rate. The observed results demonstrate that US-SC-CO_2_ exhibits superior performance compared to other processes because it produces smaller grains, increased microhardness, and improved resistance to corrosion.

The production of graphene, a novel substance that has revolutionized science and technology, was accomplished in 2004 by two British scientists [13]. Its qualities have been greatly improved, especially with regard to biocompatibility and the impacts of mechanical, heat, optical, and electrical forces [14,15]. Ahmad Raza Khan Rana and colleagues [16] prepared Ni-P-G (graphene) plating with four different compositions by adding graphene suspensions at different concentrations to electroless nickel plating. The group also looked at both the substrate’s and the plating’s resistance to scratches and compression. The outcomes showed that adding graphene to the plating increased their hardness and wear resistance. Furthermore, graphene-supported nickel plating demonstrated enhanced toughness through the prevention and removal of cracks. Using a Variable Angle Spectroscopic Ellipsometry (VASE), Marco Castriota et al. [17] examined the optical characteristics of monolayer graphene generated by chemical vapor deposition (CVD) that was transferred from a Cu substrate onto SiO_2_/Si over a broad energy range (0.38–6.2 eV). An inadvertent doping is detected, and it is discovered that the Lorentzian oscillator model of the graphene optical response matches well with the experimental results. By chemical vapor depositing graphene onto a copper foil and then the atomic layer deposition (ALD) of Al_2_O_3_ upon cu-loaded graphene, Peter Rafailov et al. [18] created Al_2_O_3_/graphene heterostructures. It was discovered that the plating had adjustable thickness, full coverage, and smooth surfaces. Using conventional techniques, the samples of these heterostructures were transferred to glass substrates, and the Al_2_O_3_ plating served as a shield during the transfer. Graphene efficiently inhibited the entrance of chemicals linked to ALD onto the surface of the Cu substrate while withstanding the development of Al_2_O_3_ ALD without causing substantial fault formation.

Graphene quantum dots (GQDs) are substances consisting of one or more sub-100 nm-thick graphene layers. Notable properties of GQDs include low toxicity, photostability, edge effects, and quantum confinement effects. Despite these features, they nonetheless possess the better mechanical, thermal, and electrical properties of graphene. There are now several biological, drug delivery, sensor, nanocircuit, and semiconductor applications being studied for them [19,20,21,22]. Zhixian Li et al. [23] prepared a Ni-GQDs composite plating by incorporating GQDs as a second-phase additive and utilizing them in nickel-based composite electrodeposition. In comparison to nickel plating, the integration of GQDs has the ability to increase the microstructure of the Ni-GQDs composite plating. The mechanical properties and corrosion resistance of the plating were significantly improved, and the grains were polished.

Pulsed electrodeposition has been shown to limit concentration polarization, improve the quality of the coated layer, and either totally remove or significantly reduce hydrogen embrittlement [24]. In addition, the coated layer has smaller grains, a denser and more uniform surface, and superior mechanical and chemical characteristics than typical DC electrodeposition [25]. The double-pulse electrodeposition process introduces a series of reverse pulse currents following the completion of a series of forward pulse currents in the plating process. The forward pulse has a long working time, while the reverse pulse has a short working time [26]. By dissolving the plating’s protruding portions with a large, brief reverse pulse current, the plating may be made more uniform and flat [27]. Reverse pulse current application can assist in spreading the plating’s thickness uniformly, speed up the oxidation of the hydrogen atoms in the plating, and mitigate the consequences of hydrogen embrittlement [28]. Meanwhile, the reverse pulse current’s dissolving effect can quickly raise the percentage of metallic ions on the cathode surface, optimizing the plating effect in the cycle that follows, decreasing the porosity of the plating, and ultimately improving the density and smoothness of the plating [29]. X. Ren et al. [30] evaluated the effects of three distinct electrodeposition techniques—double pulse, single pulse, and DC—on the microstructure and surface characteristics of composite plating. The results showed that when DC, single-pulse, and double-pulse electrodeposition procedures were used, the density and microhardness of the composite plating steadily improved while the porosity, plating speed, and grain size showed a commensurate decrease. The composite plating made using double-pulse electrodeposition showed remarkable results in terms of corrosion resistance and mechanical properties.

As there is currently no literature available comparing the processes of producing Ni-GQDs composite plating under various current sources in the supercritical state, experiments were conducted in this study to produce Ni-GQDs composite plating under DC power supply, single-pulse power supply, and double-pulse power supply. The composite plating created using three different power sources was studied in terms of its surface morphology, grain structure, wear resistance, and corrosion resistance.

In this research, we report on the production of GQDs as the second-phase additive for supercritical CO_2_ electrodeposition to create Ni-GQDs composite plating. Based on the initial experiments, the optimal parameters of the supercritical double-pulse electrolysis process were derived [27]. These were determined to be 10.5 MPa of pressure, 50 °C of temperature, 5 A/dm^2^ of forward current density, 0.25 forward pulse cycle, 0.8 A/dm^2^ of reverse current density, 0.25 reverse pulse cycle, and 1000 Hz of frequency, and an electrolysis time of 60 min. To investigate the characteristics and advantages of the supercritical electrodeposition process, a comparison is made between Ni-GQDs composite plating prepared using the DC electrolysis process, the single-pulse electrolysis process, and the double-pulse electrolysis process. To investigate the impact of GQDs on the plating, a comparison was made between the Ni-GQDs composite layer and the nickel plating created by double-pulse electrodeposition. Subsequently, the impact of four distinct processes on the plating organization, grain size, mechanical characteristics, and corrosion resistance was evaluated, and enhanced process parameters were identified. This provides a foundation for optimizing the supercritical CO_2_ double-pulse electrodeposition process and conducting further research on novel nickel-based composites.

## 2. Experimental

### 2.1. Materials

Nickel sulfate hexahydrate (NiSO_4_·6H_2_O, AR), Nickel chloride hexahydrate (NiCl_2_·6H_2_O, AR), and Boric acid (H_3_BO_3_, AR) provided by Wuxi Jingke Chemical Co. Ltd. (Wuxi, China), Sodium lauryl sulfate (C_12_H_25_NaO_4_S, CP), Citric acid monohydrate (C_6_H_8_O_2_·H_2_O), and Urea (CH_4_N_2_O) provided by China National Pharmaceutical Group Corporation Chemical Reagent Co. Ltd. (Shanghai, China). The chemical agents were used as raw materials in research without any treatment.

### 2.2. Preparation of Plating

**Figure 1:**
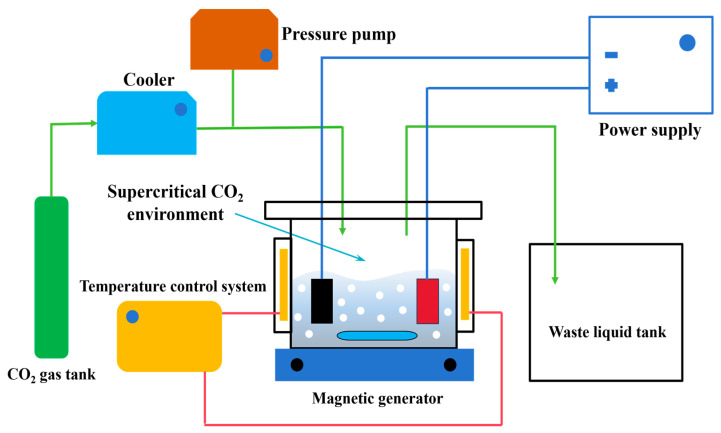
Supercritical CO_2_ fluid-assisted electrodeposition device.

Our research group independently developed a supercritical CO_2_ fluid-assisted electrodeposition device, which is shown in Figure 1. The machine uses a cooler to regulate the carbon dioxide’s temperature. Supercritical CO_2_ conditions are created by the temperature control system and pressure pump, which manage the reactor’s temperature and pressure. Throughout the experimental inquiry, several electrodeposition techniques could be compared thanks to the employment of an intelligent pulse electroplating power supply with multi-group commutation.

Experimental Methods:

(1) Preparing the second phase additive for the experiment, known as GQDs.
(2) Pretreat the electrodeposition fixture’s cathode and anode to ensure that it satisfies the experimental specifications.
(3) Configuring Watt plating solutions for electroplating.
(4) For experiments, switch on the supercritical CO_2_ fluid-assisted electrodeposition apparatus.

Experimental Steps:

(1) According to what is shown in Table 1, urea (CH_4_N_2_O) and citric acid (C_6_H_8_O_7_·H_2_O) were quantitatively weighed, mixed thoroughly with ultrapure water, and put in a high-temperature, high-pressure reactor. After being treated in a high-temperature oven, the solution containing GQDs was mixed with anhydrous ethanol and centrifuged. To make the GQDs powder, the centrifuged solid is combined with ethanol once again, cleaned, and allowed to stand, and the top suspension is removed before it is dried.
(2) This configuration uses two electrodes: a 20 × 20 mm pure nickel block for the cathode and a 20 × 20 mm copper plate for the anode. The distance between the two electrodes is 20 mm. Using 400# to 7000# sandpaper, the copper plate is polished to eliminate any oxidation layer and surface imperfections, leaving the surface mirror-like. To eliminate surface imperfections, the pure nickel block is polished using 400# and 1200# sandpaper.
(3) The formulations of the plating solutions [31] are presented in Table 2. After the following chemicals were measured out in accordance with the plating solution formula, Beaker 1 was filled with 100 mL of ultrapure water: NiSO_4_·6H_2_O, NiCl_2_·6H_2_O, H_3_BO_3_, and C_12_H_25_NaO_4_S. Once the solution has been well mixed and the magnetic stirring has been turned on, add 0.1 mL of Tergitol TMN 3 (Meryer (Shanghai) Chemical Technology Co., Ltd., Shanghai, China), a non-ionic surfactant. A certain amount of GQDs was added to Beaker 2, along with 50 mL of ultrapure water, and sonicated for 10 min to produce a homogenous aqueous solution of GQDs. After moving the mixture from Beaker 2 to Beaker 1, sonicate the mixture for a further 45 to 60 min.
(4) After turning on the magnetic stirring mechanism, fill the supercritical electrodeposition reactor with the well-mixed plating solution. Turn on the temperature control system once the reactor has been shut down. After the reaction kettle reaches 50 °C, add cooled CO_2_ gas to it [27]. For a time, maintain the pressure. After that, switch on the power supply and start the electroplating procedure.

All of the parameters used in this electrodeposition experiment are listed in Table 3. These include the inclusion of GQDs, operating pressure, electrodeposition temperature, plating time, and current setting parameters.

### 2.3. Characterization

The surface morphology and structure of the plating were investigated using the Carl Zeiss AG, Jena, Germany, SIGMA500 field emission scanning electron microscope (SEM). The plating was examined using energy dispersive spectroscopy (EDS). A Gatan 691 ion thinning device (Gatan, Pleasanton, CA, USA) was used to thin the plating to about 100 nm. Subsequently, the attachment of graphene quantum dots (GQDs) to Ni grains in the plating was seen and analyzed using an FEI Talos F200X transmission electron microscope (TEM, Hillsboro, OR, USA). Panalytical Company of the Almelo, The Netherlands’ HD-XpertPRO X-ray diffractometer (XRD) was utilized to assess the plating. The Cu-Kα radiation was used for XRD, and scanning angles varied from 5 to 80°. Following the test, the plating’s XRD spectra were examined and computed. The results were analyzed after the chemical structure of the plating was measured using a HORIBA HR Evolution Raman spectrometer (Irvine, CA, USA). The Dimenson ICTON atomic force microscope (AFM, Bruker Corporation, Billerica, MA, USA) was used to assess the plating’s surface morphology and roughness. The HXD-1000TMS digital microhardness (Shanghai Taiming Optical Instrument Co., Ltd., Shanghai, China) tester was utilized to determine the hardness of the plating. Five different plating surface areas were used for the test, and the evaluation was based on the mean hardness measurement. The experimental force was set at 200 gf.

For the plating friction and wear tests, which were carried out using the Nanovea frictional and abrasion testing machine (Nanovea Company, Irvine, CA, USA), a highly polished chromed steel bearing ball with a size of 6 mm was used. Ten N of weight, 200 rpm of rotation, and 10 min of experimentation were the experimental settings. Following the experiments, the experimental machine’s friction coefficient graphs were extracted and examined. Utilizing a Nanovea PS50 (Irvine, CA, USA) optical profiler including a 2 mm × 2 mm scanning area, 3.33 mm/s scanning rate, and 5 μm step size, the wear marks of the plating were assessed and contoured. The surface morphology of the wear traces on the plating was investigated using an SEM. The plating underwent electrochemical corrosion testing employing a Metrohm PGSTAT302N electrochemical workstation (Herisau, Switzerland). Three percent sodium chloride solution was used for the studies. Ag/AgCl (1 M) was used as a reference electrode, a platinum sheet was used as the opposite electrode, and the plating was used as the working electrode in these investigations. The dynamic potential polarization curves were tested at a scanning rate of 0.5 mV/s and a voltage range of −0.2 V to 0.2 V. With a sinusoidal amplitude of 10 mv and a frequency range of 0.01 to 10,000 Hz, the electrochemical impedance spectra were observed. At the conclusion of the experiments, the findings were examined. The plating was immersed in a solution of 3.5% sodium chloride over 120 h for the corrosion experiments. After the corrosion, the plating’s corrosion morphology was examined and examined using an SEM.

## 3. Results and Discussion

### 3.1. Microstructure Analysis of GQDs

Figure S1 shows physical and transmission electron microscopy (TEM) images of graphene quantum dots (GQDs). The resulting GQDs are in the form of spherical or near-spherical particles with a particle size of about 6 nm.

### 3.2. Effect of Different Electrodeposition Processes on Plating Microstructure

**Figure 2:**
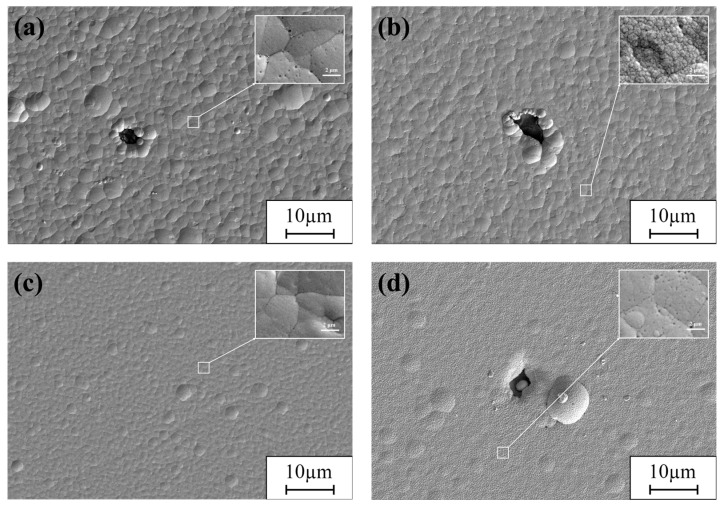
SEM photos of Ni-GQDs composite plating and Ni plating made using various electrodeposition techniques. (**a**) Ni-GQDs-I; (**b**) Ni-GQDs-II; (**c**) Ni-GQDs-III; (**d**) Ni plating.

The supercritical CO_2_ conditions used to create the Ni-GQDs composite plating using various electrodeposition techniques are depicted in Figure 2 through SEM pictures. Examining the figure’s complexity up close reveals tiny flaws in the plating layer. For this reason, during the supercritical electrodeposition process, it is unavoidable that some of the supercritical fluid will be deposited on the plating layer, resulting in tiny insulating patches. The holes that arise from this source, however, are so small that they hardly even register on the plating and are only visible at extremely high magnification. Figure 2a displays the composite plating of Ni-GQDs-I obtained using direct current (DC) electrodeposition. The Ni-GQDs-I composite plating’s surface morphology is noticeably asymmetrical, as seen in Figure 2a, with several uneven protrusions and apparent pinholes. The single pulse electrodeposition-produced Ni-GQDs-II composite plating is shown in Figure 2b. The surface quality of the Ni-GQDs-II composite plating has improved as surface protrusion decreases in comparison to the DC electrodeposited layer. On the plating’s surface, there are still tiny pits and holes at the grain boundaries, though. Figure 2c depicts the exterior of the Ni-GQDs-III composite plating produced by double pulse electrodeposition. The Ni-GQDs-III composite plating’s surface has much improved, displaying a reduction in grain size and no pinholes. The Ni plating generated by double-pulse electrodeposition is shown in Figure 2d. Even though the surface of the Ni plating is usually smooth, pockmarks and pinholes frequently develop.

**Figure 3:**
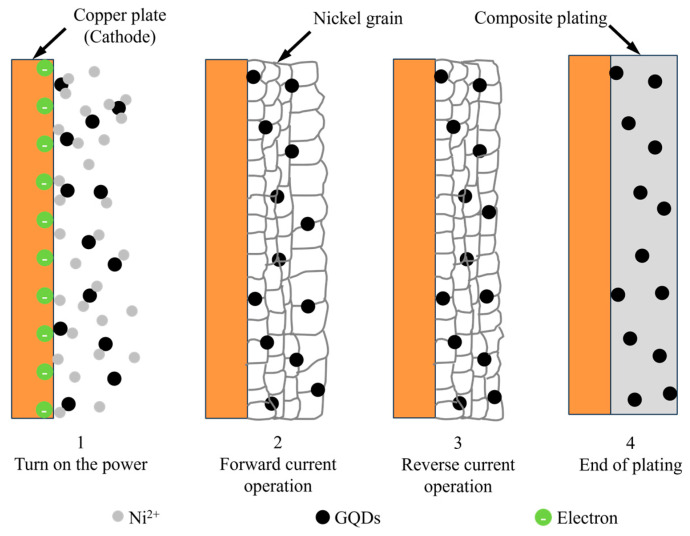
Co-electrodeposition mechanism of Ni and GQDs.

Figure 3 shows the mechanism diagram for the co-deposition of Ni and GQDs. At the beginning of the electrodeposition process, the GQDs attract Ni^2+^ in the plating solution, which renders them positively charged and causes them to deposit slowly on the surface of the components that require plating because of poor adsorption at the cathode. When the power supply is turned on, strong adsorption takes place, leading to the co-deposition of Ni^2+^ and GQDs on the cathode’s surface as a result of magnetic swirling and an electric field force [32]. The primary stages 1, 2, and 4 of Figure 3—the power supply turning on, the power supply operating, and the end of plating, respectively—are encountered in DC electrodeposition and single-pulse electrodeposition. The distinction is that single pulse electrodeposition functions indirectly, whereas DC electrodeposition operates continuously. The reverse pulse function of the double-pulse electrodeposition can further purge the plated surface of contaminants and improve the grain. Turn on the pulse power supply and begin plating, as illustrated in Figure 3. When the forward current is operating, the composite plating layer is continuously deposited; after a while, the forward current is switched off and the reverse current begins to operate, giving the impression that the plating layer is partially dissolved, the outer layer of grains is beginning to refine, and the impurities within the plating layer are dispersed and released.

Because of the continuous current flow, constant consumption of Ni^2+^ at the cathode, and temporary lack of Ni^2+^ replacement, concentration polarization occurs during the electrodeposition test when a DC power source is used [33]. At this moment, the charge and mass transfer processes on the cathode surface are inhibited, and the rate of cathodic deposition is reduced. In close proximity to the cathode, the water solvent electrolytes in the plating solution simultaneously convert H_2_O into H^+^ and O^2−^. Near the cathode, H^+^ is subsequently converted to H_2_, which adheres to the surface to create an insulating patch. The presence of H_2_ causes pinholes to form at the conclusion of plating, which disturb the surface structure of the plated layer and appear as holes, as seen in Figure 2a. In addition, DC deposition produces a stronger electric field, faster grain development, and higher current efficiency—all of which might cause the Ni-GQDs-I composite plating to have coarse grains, poor flatness, and poor surface quality. The single pulse electrodeposition approach offers superior processing advantages over DC electrodeposition because it has a certain duty cycle value that is excellent for minimizing the concentration polarization induced by DC electrodeposition. In single-pulse electrodeposition, the concentration of Ni^2+^ at the cathode gradually returns to its initial level upon termination of the forward current. This increases processing efficiency by guaranteeing that an adequate concentration of ions is available for subsequent electrodepositions. Furthermore, the instantaneous high-current features offered by the single-pulse power supply may significantly improve the plated layer’s surface quality and fine-tune its grain size. As shown in Figure 2b, the Ni-GQDs-II composite plating has a cauliflower head form in terms of microscopic morphology, and it has a larger surface flatness and superior surface quality than the Ni-GQDs-I composite plating. However, due to the increased current density of the forward pulse, ions near the cathode were rapidly depleted, and cation replenishment during the pulse-off phase was slower than cation depletion during the pulse-on phase. This leads to a concentration polarization phenomenon [34] that exacerbates the process of hydrogen precipitation even further. At this moment, hydrogen adheres to the surface of the electrodeposited cathode layer, preventing certain places from deposition of nickel ions.

The surface quality of Ni-GQDs-III composite plating created by double pulse electrodeposition is superior to that of Ni-GQDs-I and Ni-GQDs-II. When using double-pulse electro-deposition, the forward current is run while the Ni^2+^ and GQDs from the plating solution are co-deposited on the surface of the component that is to be plated at the cathode. After some time, the reverse duty cycle and current start to operate, and the forward current is shut off. After that, the part that has to be plated serves as the anode, and some dissolution occurs to get rid of any imperfections that could have shown up on the composite plating layer. The Ni^2+^ concentration in the plating solution gradually increases throughout the process, with an optimum concentration of Ni^2+^ around the part to be plated. Hydrogen precipitation and concentration polarization effects from the forward pulsed current density are reduced in successive electrodeposition cycles [35]. The double-pulse electrodeposition method therefore yields a plated layer with a tiny particle size, a smooth surface, and no obvious defects.

The Ni plating produced by the double pulse electrodeposition process exhibits inferior surface quality due to the absence of a second-phase additive. The introduction of an additional phase component to the plating process can result in the formation of additional nucleation sites on the plating’s surface. This may impede the growth of grains and facilitate the refinement of grains [36]. The interstitial gap may decrease as a consequence of the grains being closer together as a result of smaller grain development. Nevertheless, the grains enlarge and the gaps between them increase due to the absence of GQDs in the Ni plating. As a result, there are more pockmarks and pinholes on the plating’s surface, which seems less organized.

The content and dispersion of GQDs in Ni-GQDs composite plating formed by the DC electrodeposition technique, single-pulse electrodeposition method, and double-pulse electrodeposition method were investigated using EDS planar scanning [37]. An EDS scan of every composite plating is presented in Figure S2. The Ni-GQDs-I composite plating created using the DC electrodeposition process has a relatively low amount of carbon components—5.7%. In the composite plating Ni-GQDs-II made using the single-pulse electrodeposition method, the proportion of carbon components rises to 7.2%. In Ni-GQDs-III composite plating created by double pulse electrodeposition, element C makes up a higher proportion (8.8%). The distribution of the C and Ni components inside the Ni-GQDs-III composite plating is shown in Figure S3, which demonstrates that the elements C and Ni are evenly distributed throughout the plating and that the GQDs and Ni are scattered without agglomeration.

During the double-pulse electrodeposition process, a large number of GQDs were deposited into the plating, according to the EDS planar scanning data [38]. The surface form of the Ni-GQDs-III composite layer was improved by the even and dense distribution of nickel grains and GQDs inside it. Studies have indicated that the plating layer’s surface quality can be improved to a more acceptable level by adding GQDs.

**Figure 4:**
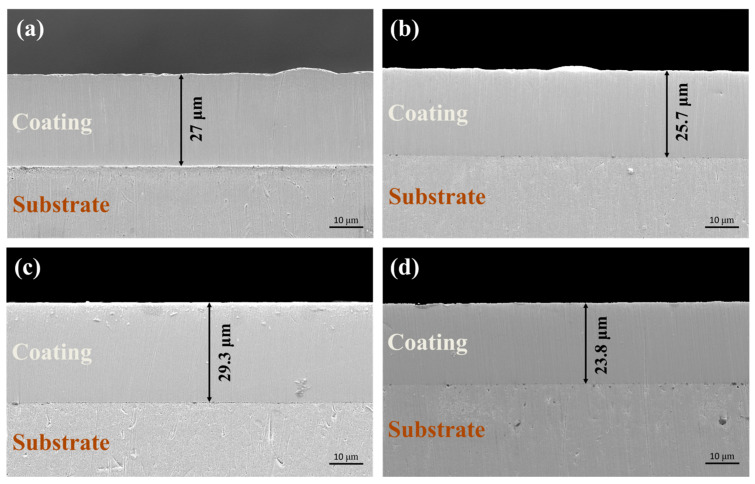
Cross-sectional SEM images of Ni-GQDs composite plating and Ni plating created using several electrodeposition methods: (**a**) Ni-GQDs-I; (**b**) Ni-GQDs-II; (**c**) Ni-GQDs-III; (**d**) Ni plating.

The strong connection between the substrate and plating is seen in Figure 4, which displays the cross-sectional SEM images of the four platings. The thickness of each plating is 27.0 μm, 25.7 μm, 29.3 μm, and 23.8 μm, respectively. Tiny fractures form at the bonding site because of the hardness differential between the substrate and plating materials during the grinding process. Rather than insufficient plating bonding, this is brought on by the impact of the abrasive particles. Simultaneously, we saw that the Ni-GQDs composite films produced by the double-pulse power supply had improved cross-sectional homogeneity, with comparatively flat films that were free of noticeable bumps. Raised grains are clearly visible in the cross-sections of the Ni-GQDs-I and Ni-GQDs-II composite plating, although the latter’s bumps are less noticeable. This indicates that because the single-pulse electrodeposition approach may reduce plating defects and polish grains, it produces composite plating with superior surface morphology. The pure Ni layer has a comparatively flat cross-section because of the double-pulse power source’s deposition characteristics.

The fact that the DC electrodeposition composite plating has a greater thickness indicates that the composite plating made with this power source is very effective. It can also be made with a lower cost, simpler operation, and fewer parameter variations. When a duty cycle parameter is applied, the single-pulse electrodeposition process’s current fluctuates between on and off, resulting in instantaneous high-current characteristics. This can be a great way to improve the microstructure, refine the grain size, deposit more GQDs, and raise the organization of the plating’s grains. When a single-pulse power source is employed for electrodeposition, grain density increases, and well-restrained grain growth is accomplished. As a result, the thickness and grain development rate of the plating is inherently lower than those of the Ni-GQDs-I composite plating, which was driven by a DC source. Based on a single-pulse power supply, double-pulse electrodeposited technology introduces reversal duty cycles and reversed current densities. In addition to ensuring that a concentration of Ni^2+^ is present close to the cathode to minimize the formation of concentration polarization phenomena in the plating solution, reverse current can effectively remove impurities and defects formed during the plating process on the exterior of the plated film. As a result, the double-pulse electrodeposition process yields a composite plating layer with improved surface shape, increased grain fineness, and increased GQDs content. These elements may result in a thicker plating layer overall and a larger density of nickel grains perpendicular to the direction of the plating layer, as well as more nucleation sites for the formation of nickel grains. As for the pure nickel plating prepared by the double-pulse power supply, due to the absence of GQDs in the plating solution, the produced plating does not contain any GQDs, which cannot provide nucleation sites in the plating layer, and the deposition current is only used for Ni^2+^ deposition and grain growth. However, the double-pulse power source that was utilized to create this plating efficiently removes imperfections like contaminants from the plating’s surface and refines the plating grains when the reverse current is activated. Ni plating thus has the shortest thickness. This demonstrates the dual-pulse power supply’s advantage even further.

**Figure 5:**
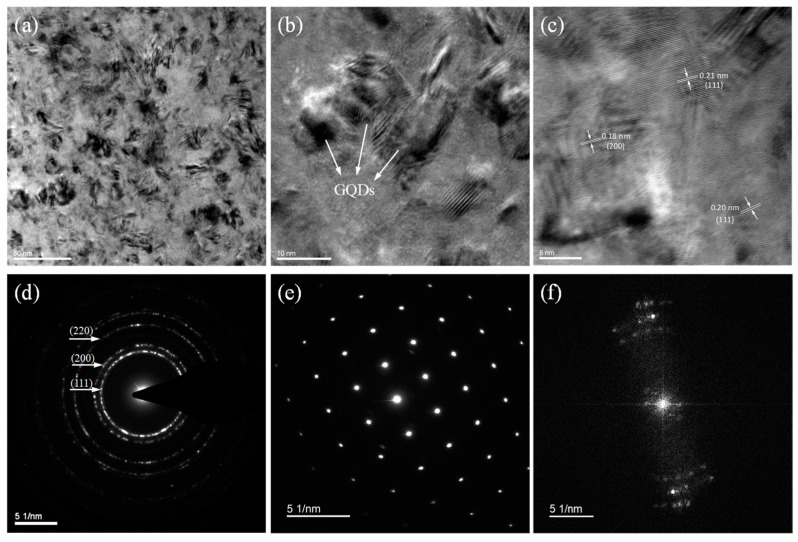
TEM images of Ni-GQDs-III composite plating (**a**–**c**); (**d**) SAED diagram for plating; (**e**) SADP image of the plating; (**f**) FFT image of the plating.

By using TEM to analyze the microscopic structure of the Ni-GQDs-III composite layer, a greater understanding of the spatial distribution of GQDs throughout the plating was achieved [39]. Figure 5 illustrates a TEM image of the plating. As shown in Figure 5a, the total grain size of nickel is constant and has already reached the nanoscale, despite the large diameter variation among individual grains. There are clear grain boundaries between the nickel grains in the plating, and the GQDs are uniformly dispersed throughout the plating. Based on the high magnification TEM data presented in Figure 5b, the concentration of GQDs particles in the plating indicates that the GQDs have been deposited into the plating efficiently. In addition, a thin nickel film exists in the GQDs’ surface layer, a tiny hump on the winding of the GQDs, and a visible particle interface. This advantageous interface combination facilitates the loading pressure transfer between the substrate and the plating, improving the mechanical characteristics of the plating. The Ni-GQDs composite plating is seen at a higher magnification in Figure 5c. The d-distance values of the plating are shown and displayed in the figure; they coincide with the crystalline surface of ordinary nickel powder. Crystal planes (111), (200), and (220) are visible in the secondary electron diffraction (SAED) pattern of the plating, as shown in Figure 5d [40]. Based on this, the plating seems to have a face-centered cubic structure. The plating’s SADP diagram is displayed in Figure 5e. The symmetrical distribution and regular arrangement of the white spots in the combination of particle plating with respect to the center of gravity may indicate that the Ni particles have a single crystal structure without subgranular boundaries. This agrees with what the XRD charts showed. Figure 5f displays the FFT image of the Ni-GQDs-III composite plating. The results are consistent with the electron diffraction image from the TEM shown in Figure 5d.

A planar EDS scan was performed using TEM to determine the elemental composition of the composite plating of Ni-GQDs. Figure S4 displays the scan findings, and Figure S4a displays the surface topography of the plating. The nickel and carbon components of the plating are distributed as seen in Figure S4b,c. The elements C and Ni are evenly spaced throughout the plating, as shown in Figure S4b,c. This agrees with the EDS scan findings from the SEM. This data shows that the Ni grains and GQDs were dispersed uniformly throughout the plating and that the GQDs were effectively deposited in the plating. The proportion of carbon components in the plating is presented in Table 4 and Figure S4d. It reveals that the mass and atomic percentages of the C element in the plating are, respectively, 2.34% and 10.5%. This data shows that GQDs have been effectively placed in the plating.

### 3.3. Effect of Different Electrodeposition Processes on XRD

**Figure 6:**
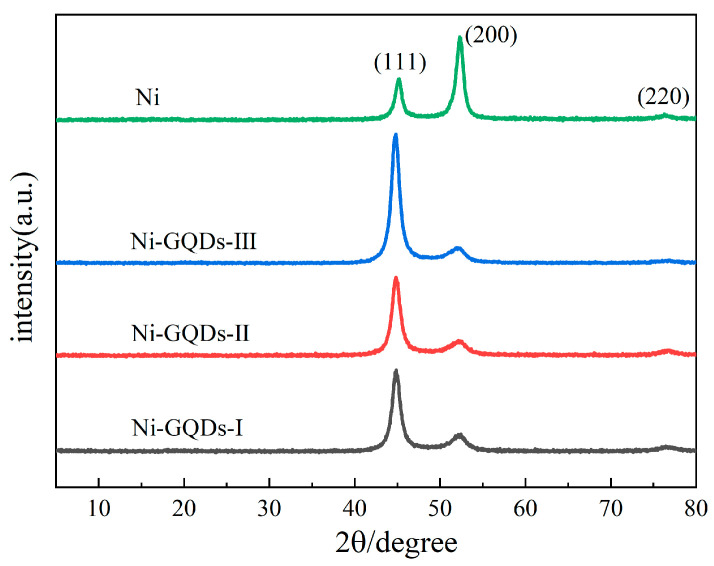
XRD patterns of plating made using various electrodeposition techniques.

Figure 6 depicts the XRD patterns of the plating produced in supercritical carbon dioxide (SC-CO_2_) settings by different electrodeposition techniques. As can be shown in Figure 6, the plating created using different electrodeposition techniques exhibited diffraction peak angles of around 45°, 52°, and 76°. The (111), (200), and (220) crystal planes of Ni grains are represented by these angles, in that order. According to this, there was a face-centered cubic crystalline structure of Ni in the plating [41], which is consistent with the TEM results from the SAED perspective. Compared to the (200) and (220) textures, the (111) facet of the Ni-GQDs composite plating created by DC electroplating, single-pulsed electroplating, and double-pulsed electroplating shows stronger diffraction peaks. When the Ni plating was created by double-pulse electrodeposition, the (200) surface exhibited a higher diffraction peak intensity than the (111) and (220) textures. This is due to the fact that the uniform distribution of GQDs in the supercritical CO_2_ environment increases the number of nucleation sites on the plating and inhibits the formation of nickel crystals on the (200) crystal plane. Ni crystal formation is inhibited during electrodeposition, which promotes atom movement to the most stable sites. Consequently, the (111) facet of Ni crystals is the favored orientation in Ni-GQDs composite plating [42]. The (200) texture is the preferred orientation of nickel crystals in the nickel plating layer when GQDs are not introduced into the plating during the electrodeposition process [43]. This is because the absence of GQDs in the Ni plating results in the absence of GQDs at the Ni plating’s defect sites to absorb boron and SDS in the plating solution used to optimize deposition.

The half-peak widths and diffraction angles of the plating’s reflection peaks, which are produced by the different electrodeposition techniques, are included in the XRD test findings. Furthermore, the grain size of the plating may be estimated using the Debye-Scherrer formula [44]. Below is the formula for your reference.

(1) $$D=\frac{K\lambda }{\beta COS\theta }\cdot \frac{180}{\pi }$$

Equation (1) states that the diffraction angle is *θ* the width of the reflection peak at 1/2 high is *β*, the X-ray wavelength (*λ*) is 0.154056 nm, and the Scherrer constant *K* is 0.89. Equation (1) was used to determine the plating’s grain size, and the outcomes are exhibited in Table 5.

Based on the XRD detection results, the diffraction intensity of each peak was obtained, and Equation (2) was utilized to compute the crystalline surfaces’ relative texture coefficient.

(2) $$\mathrm{RTC}\left(\mathrm{hkl}\right)=\frac{I_{s}\left(hkl\right)/I_{0}\left(hkl\right)}{\sum_{1}^{n}I_{s}\left(hkl\right)/I_{0}\left(hkl\right)}\times 100\%$$

where *I*_0_(*hkl*) is the reflection peak strength of the crystal peaks from the conventional Ni powders and *I_s_*(*hkl*) is the reflection peak intensity for the crystal peaks from the manufactured Ni-GQDs composite films and Ni plating. The results of the relative texture coefficient (RTC) of each crystalline surface calculated according to Equation (2) are shown in Table 5.

Table 5 shows how, for different electro-deposition process parameters, the Ni-GQDs composite plating’s grain size gradually decreases. In contrast to the Ni plating created by double-pulse electrodeposition, which had bigger grains (6.88 nm), the samples with the Ni-GQDs-III composite layer had reduced grain sizes (4.58 nm). The grain size of the Ni-GQDs-III composite plating is 33.4% smaller than that of the Ni plating. This implies that reducing the grain size by plating with GQDs is an effective technique. Ni (111) crystal plane growth is the direction in which the grains of the Ni-GQDs-I, Ni-GQDs-II, and Ni-GQDs-III composite plating gradually grow; in contrast, the Ni (111) texture development tendency is more prominent in the Ni-GQDs-III composite plating made using the double-pulse electrodeposition method. This indicates that the plating’s nickel atoms could grow more consistently and that the double-pulse electrolysis process is better at modifying the plating grain’s development. Since the absence of GQDs in the Ni plating tends to promote the Ni (200) crystal orientation, it appears that the presence of GQDs influences the preferred orientation of the Ni grains in the plating.

Continuous electrodeposition causes an ongoing ion depletion at the cathode when DC electrodeposition is utilized to create Ni-GQDs composite coatings. Concentration polarization happens at this stage because the rate of ion deposition is greater than the rate of ion replenishment. As a result, Ni^2+^-induced GQDs deposition is impeded to the point where the plated layer’s GQDs content drops and the number of nucleation sites reduces. Consequently, unlike with pulse plating, the selective orientation of the nickel (111) surface is not readily apparent, and nickel grain development is not easily inhibited.

Single-pulse electrodeposition yielded a smaller grain size while producing Ni-GQDs composite plating than DC electrodeposition did. With the deposition of more GQDs in the plating, the Ni growth mode gradually transitions to the Ni (111) texture. The plating grains are refined further by the single-pulse electrodeposition technique’s transient high-current deposition characteristic.

Reverse pulse currents added to the double-pulse electrodeposition process may efficiently remove surface flaws such as burrs and bumps and raise the concentration of Ni^2+^ in the cathode. The next electroplating cycle benefits from using a higher density of forward pulse current since it speeds up nucleation relative to crystal development. More GQDs are applied to the plating at the same time, which reduces grain size, slows down grain formation, and refines the grain structure [45].

Compared to coatings created using other methods, it was discovered that Ni plating produced using the double pulse electrodeposition approach had larger grains and less refined grains. The absence of GQDs, which offer nucleation sites to restrict the growth of nickel grains, causes the grain growth of nickel to take on nickel (200) texture, with bigger grain sizes and less refined grain.

### 3.4. GQDs Characterization in Plating Made Using Various Electrodeposition Processes

The Raman spectrum test results of the Ni-GQDs composite plating, which was made with powdered GQDs and various electrodeposition techniques, are shown in Figure S5. The absence of GQDs in the Ni plating resulted in the test results not displaying the D and G peaks. Consequently, the Raman spectral test result for the Ni plating was not present in the Raman spectrogram. Table 6 displays the positions and intensities of the D and G peaks in the Raman spectrogram as well as the correlation between their intensity levels. It has been demonstrated that the D peak’s intensity indicates the percentage of flaws, structural abnormalities, and amorphous parts present in the sample. On the other hand, the material’s lattice integrity and number of layers are indicated by the strength of the G peak [46]. Consequently, more flaws in the sample’s C atom crystals are indicated by a larger D peak intensity value. Conversely, a greater G peak intensity value suggests that the sample has a greater number of complete C atom crystals. Thus, the quality of GQDs is determined by the ratio I_D_/I_G_ of the intensity values for the D-peak and G-peak. Higher-grade GQDs might be indicated by a lower I_D_/I_G_ number [47].

The D and G peaks of the GQDs and Ni-GQDs composite plating are located in identical locations, as shown in Figure S5 and Table 6. According to the test results, the powder may have more faults per unit area since the GQDs had the highest I_D_/I_G_ value. After DC electro-filtration, the Ni-GQDs composite plating’s I_D_/I_G_ values decreased, suggesting a decrease in GQDs flaws and an increase in their quality. This might be explained by the effective improvement of GQDs integrity achieved in supercritical CO_2_ conditions using electro-filtration.

Due to the transient high-current characteristic of pulsed electrophoresis, the single-pulse electrophoresis approach enhanced the integrity of the inner GQDs, decreased plating defects, and fine-tuned the plating grain size. The reverse pulse current may effectively remove impurities and contaminants from the plating’s surface when a double-pulse electroplating current is used. This enhances the plating’s surface quality, optimizes the GQDs’ internal structure, and reduces defects. As a result, the double pulse electrodeposition method yields Ni-GQDs composite plating with improved surface quality, a decreased I_D_/I_G_ ratio, and a noticeable drop in flaws.

### 3.5. Effect of Various Electrodeposition Methods on Mechanical Properties of Plating

#### 3.5.1. Effect of Different Electrodeposition Processes on Microhardness of Plating

**Figure 7:**
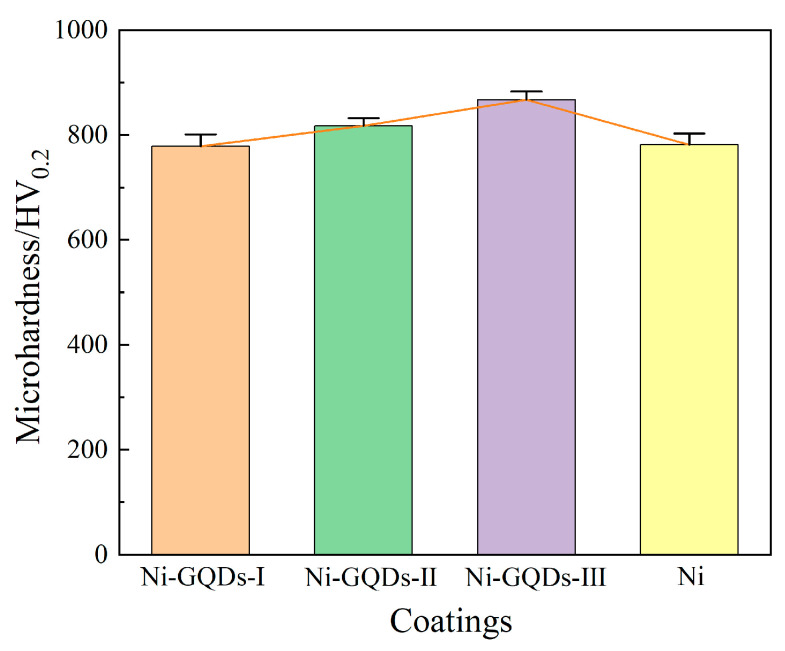
Microhardness diagrams of plating made using various electrodeposition techniques.

Figure 7 displays the microhardness curves of the plating produced by the different electrodeposition methods. As illustrated in Figure 7, the mean hardness values of the four plating exhibited variation according to the electrodeposition process, with a stable mean value of 778.5 HV, 817.3 HV, 867.2 HV, and 781.2 HV, respectively. The Ni-GQDs-I composite plating manufactured by DC electrolysis was found to have a lower hardness than the Ni-GQDs-II composite plating plated by single pulsed electrolysis, which had a slightly higher hardness. The Ni-GQDs-III composite plating, on the other hand, made using the double-pulse electrolysis method demonstrated a notable 11.4% increase in hardness, reaching a maximum hardness of 867.2 HV.

The following equation illustrates how the grain size might be responsible for the difference in hardness, based on the Hall-Petch relation [48]:

(3) $$HV=HV_{0}+K/\sqrt{d}$$

Grain size (*d*) and experimental constants (*HV*_0_ and *K*) are given in Equation (3). Thus, the plating’s hardness and grain size are inversely correlated, the smaller the grain size, the harder the plating.

The constant working current in DC electrodeposition causes the plating grain to develop fast and big, making it harder to resist the dislocation motion caused by a higher loading force [49]. Moreover, the Ni-GQDs-I composite plating has a low microhardness value due to its reduced GQDs content and the plated layer’s decreased resistance to external stresses. The plated layer’s grain size is reduced, the grain-to-grain connection is reinforced, the resistance to external loads is raised, and the propensity towards plastic deformation is lessened when electrodeposition is carried out with a single pulse power source. The microhardness value of the Ni-GQDs-II composite plating rises as a result.

The double-pulse electrodeposition method utilized to produce the Ni-GQDs-III composite plating significantly improves the quality of the plated layer. Reverse pulse current reduces the likelihood of the phenomenon of concentration polarization by turning the cathode into an anode, removing surface flaws like burrs and dents on the plated layer, and restoring the concentration of cations close to the cathode. In addition, more nucleation sites for Ni^2+^ development are supplied by encouraging the deposition of more GQDs into the plating layer by double-pulse electrodeposition, and the nucleation rate of grains is higher than the growth rate of grains. As a consequence, the Ni-GQDs-III composite plating reaches its maximum microhardness value, producing a prepared composite plating layer with tiny grain sizes, high grain densities, and enhanced resistance to dislocation motion.

In pure nickel plating produced using the double-pulse approach, there are no GQDs added to the plating solution or GQDs in the layer to provide nucleation sites to restrict the development of nickel crystals during electrodeposition. Consequently, the plating layer experiences an increase in grain size, a rise in intergranular voids, and a weakening of its capacity to withstand the dislocation motion brought on by the loading force, ultimately leading to a drop in the plating layer’s hardness.

#### 3.5.2. Effect of Different Electrodeposition Processes on Plating Surface Roughness

Table 7 displays the Ra and Rq of the composite plating of Ni-GQDs, whereas Figure S6 shows the AFM images of the plating generated by the different electro-deposition methods. Figure S6 illustrates how the electrodeposition technique affected the mean roughness. More mean roughness was observed in Ni-GQDs-I composite plating made by DC electroplating than in Ni-GQDs-II composite plating made by single-pulse electroplating. It was also discovered that the surface of the Ni plating created by the double-pulse electroplating process was rougher. The double-pulse electroplating method yielded a composite plating of Ni-GQDs-III with a roughness of 47.3 nm micrometers, which is a lesser degree. Compared to the other plating, there was a noticeable 54.1% reduction in roughness.

Due to the constant nature of the positive current in DC plating, the cathode is prone to consuming a significant amount of ions, ending in severe polarization. Concentration polarization leads to imperfect surface characteristics and subpar plating outcomes. Furthermore, the current electrolyzes water, forms hydrogen precipitates, and produces bubbles on the plating’s surface due to concentration polarization. As can be seen in Figure 2a, surface flaws such as bumps and pinholes cause the plating surface to be substantially rough and lack appropriate flatness.

The ion concentration of the cathode was recovered during the pulse-off period in a single-pulse deposition. The single-pulse electrodeposition approach outperforms the DC electrodeposition approach, as can be demonstrated. It follows that the Ni-GQDs-II composite plating has less roughness than the Ni-GQDs-I composite plating [50]. The cathode undergoes faster ion depletion when a higher current density is used in the forward pulse, which keeps the cathode’s ion concentration from fully recovering during the falling pulse phase. At this time, concentration polarization takes place, increasing the electrode’s surface area and decreasing its efficiency. Bubbles form at the cathode as a result of concentration polarization-induced increase in hydrogen precipitation. This results in pinholes and pockmarks on the plating’s surface during electrodeposition. The plating is rougher on the surface than the Ni-GQDs-III composite plating, as Figure 2b illustrates. The uneven plating is to blame for this.

In the double-pulse electrodeposition technique, the reverse pulse current can correct plating surface flaws and raise the ion concentration at the cathode. This leads to a significant decrease in concentration polarization and hydrogen precipitation reactions and an enhancement of the electrodeposition effect. Figure 2c illustrates the plating surface’s density and flatness, which are free of visible defects. Furthermore, the smaller grains mean that there are fewer spaces between them, which reduces the overall unevenness of the plating.

When double pulse electrodeposition is used to create nickel plating, the plating lacks GQDs, which would serve as nucleation sites. Grain development is enhanced because the plating layer lacks second-phase additives that would normally restrict grain growth [51]. This led the nickel plating to have bigger granules and greater spaces between the grains, which decreased the plating’s surface quality. The plating has various defects, such as dents and pinholes, and its surface is a little rough, as shown in Figure 2d.

#### 3.5.3. Effect of Different Electrodeposition Processes on the Resistance to Wear of Plating

**Figure 8:**
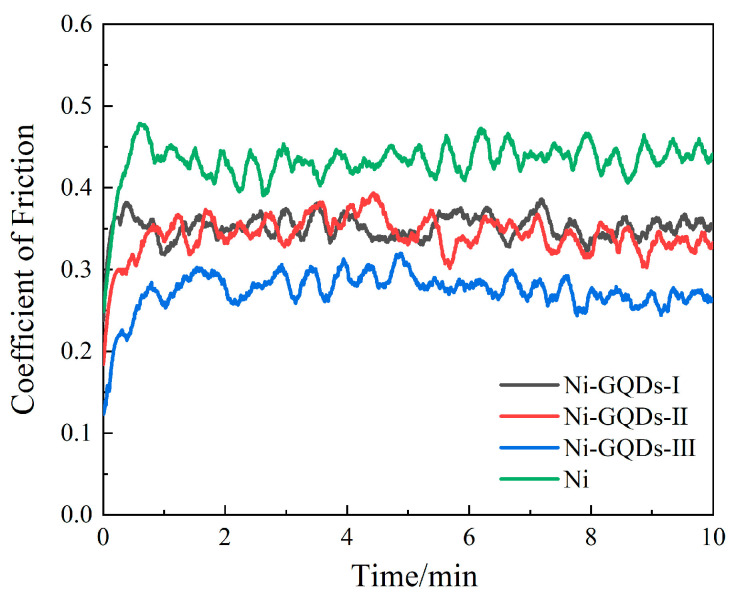
The friction coefficients of plating made using various electrodeposition techniques.

The coefficient of friction for plating produced using various techniques is illustrated in Figure 8. Each plating layer exhibits a variable friction coefficient over time. The initial fluctuations observed in the friction coefficient curve are primarily attributed to the removal of uneven regions on the plating’s surface. Due to the limited contact area between the steel ball and the plated surface, along with the inherent irregularities of the plating, significant variations in the friction coefficient are evident when the two surfaces first make contact. Stress concentrations and plastic deformation therefore occur on the plating’s surface. The friction coefficient stabilizes after the plating surface is smooth and level [52]. The friction coefficients of the composite plating (Ni-GQDs-I, Ni-GQDs-II, Ni-GQDs-III, and Ni) finally settle at 0.36, 0.33, 0.26, and 0.44, respectively. It is well known that plating with a lower coefficient of friction exhibits increased wear resistance [53]. The Ni-GQDs-III composite plating has a 41% lower coefficient of friction than the other plating, demonstrating its superior wear resistance. The low wear resistance of Ni plating, Ni-GQDs-I composite plating, and Ni-GQDs-II composite plating is shown by their higher friction coefficients.

**Figure 9:**
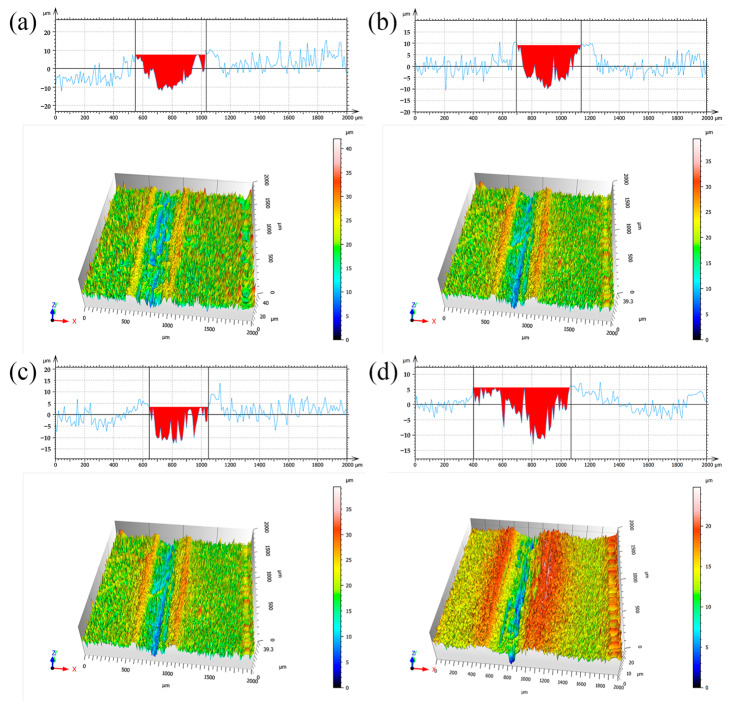
Cross-section and 3D morphology of the wear traces of the plating prepared by various electrodeposition processes; (**a**) Ni-GQDs-I; (**b**) Ni-GQDs-II; (**c**) Ni-GQDs-III; (**d**) Ni.

Figure 9 shows the sectional area and three-dimensional morphology associated with the abrasion traces on the plating of various electrodeposition processes. Table 8 displays the values of cross-sectional area, volumetric wear, and maximum depth of the wear indications on the plating. As seen in Figure 9 and Table 8, the wear traces on the Ni-GQDs-III composite plating produced by the double-pulse electrodeposition technique are less severe and have a smaller cross-sectional area than the wear traces on the plating produced by the other electrodeposition technologies. The volumetric wear rates of the Ni-GQDs-III composite plating were 64.2%, 67.0%, and 72.4% of the Ni-GQDs-I, Ni-GQDs-II, and Ni plating, respectively, based on the data. This indicates a noteworthy decrease of 27.6%. The Ni-GQDs-III composite plating outperforms other coatings in the friction wear tests in terms of wear resistance. This is demonstrated by the wear mark’s decreased cross-sectional area, shallow depth, and small wear volume created [54].

During friction and wear testing, harder steel balls may cause a plow mark phenomenon on the plating surface, which might partly destroy the plating [55]. The amount of the second phase additive indicates the number of solid lubricating places in the plating; the more solid the friction reduction points, the higher the friction reduction [56].

**Figure 10:**
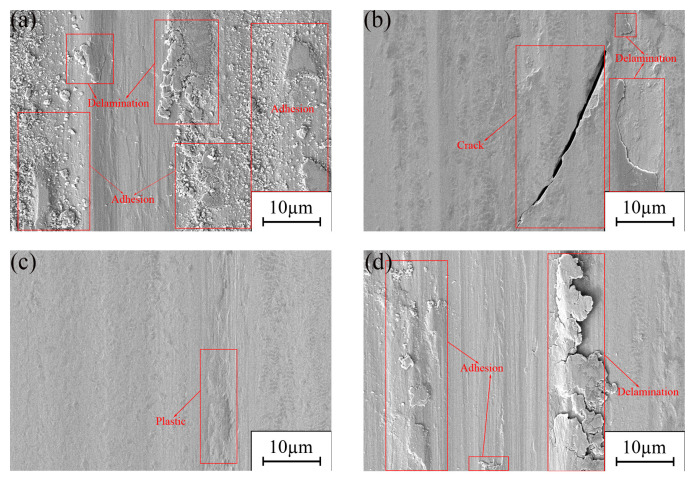
SEM image of abrasion marks on Ni-GQDs composite plating and Ni plating; (**a**) Ni-GQDs-I; (**b**) Ni-GQDs-II; (**c**) Ni-GQDs-III; (**d**) Ni.

The DC electrodeposition approach yielded a Ni-GQDs-I composite plating with coarser grain size and larger intergranular voids [57]. The plating has a low microhardness rating and a high surface roughness value. Furthermore, fewer solid friction reduction spots and a weaker plating’s shear resistance during frictional contact result from the plating’s reduced GQD concentration. Consequently, there is little wear resistance. According to Figure 10a, it can be seen that the abrasion mark morphology of the composite plating is poor, the plowing phenomenon produced by the steel ball on the plating is more serious [58], and there are a large number of adhesive wear and surface defects [59].

The Ni-GQDs-II composite plating with minuscule grain size, compact plating structure, and satisfactory surface morphology was produced by the single pulse electrodeposition technique. There are more solid lubrication spots in the plating due to the increased number of GQDs. When two plates come into contact with one another, the wear resistance of the plate increases and the coefficient of friction and frictional resistance decreases [60]. The wear marks on the Ni-GQDs-II composite plating are smoother and of greater quality, as can be seen from Figure 10b, even if there is fracture and separation. This plating has superior wear quality than Ni-GQDs-I composite plating. The excellent wear resistance of Ni-GQDs composite plating created using the single pulse electrodeposition method is demonstrated.

The Ni-GQDs-III composite plating produced by double-pulse electrodeposition has the best wear resistance. The smooth surface of the plating is free of apparent defects. Steel ball plowing impact is minimal, plating grain refinement is high, grain size is small, and hardness is high. The larger carbon content of the plating means that there are more GQDs available to provide the plating with strong friction reduction sites, which reduces the shear pressures generated during friction. The abrasion marks seen in Figure 10c exhibit excellent surface quality, including a smooth surface devoid of any extra imperfections and minimal plastic flow. It illustrates how the double-pulse electrodeposition-created Ni-GQDs composite plating has extraordinary wear resistance.

With coarse grain size, poor surface morphology, and weak resistance to dislocation movement, pure nickel plating is produced via the double-pulse plating technique. The main cause of friction, as the plating is devoid of GQDs, is the metal’s capacity to tolerate frictional shear forces when they occur. Multiple adhesions and surface imperfections cause the abrasion marks in Figure 10d to have poor surface quality. It illustrates how the plating layer is more vulnerable to shear stress and the steel ball has a stronger bead-plowing impact during frictional contact [61]. It implies that the pure nickel plating produced by the double pulse electrodeposition process has insufficient wear resistance.

**Figure 11:**
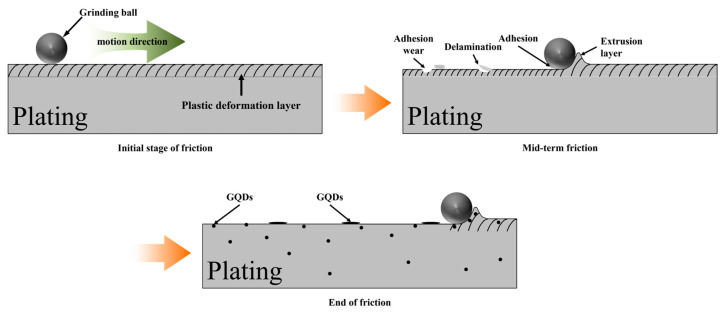
Mechanism diagram of the friction experimental process of Ni-GQDs composite plating.

The friction and wear test mechanism schematic for the composite plating is shown in Figure 11. The abrasive ball and the plating layer are in contact during the first stage of friction, which is characterized by sharp fluctuations in the friction coefficient and a certain plastic deformation of the plating layer’s surface. When the surface of the plating layer deteriorates over time, the friction coefficient gradually decreases and finally approaches a constant value. Adhesive wear and plated layer loss can occur in addition to residual plastic flow and some surface damage to the plated layer. In friction testing, a plating’s wear resistance is positively connected with its grain size. Smaller grains have better inter-grain connections, more grains that resist external deformation per unit area, fewer loading pressures applied to individual grains when an external load is applied, and better plating resistance against dislocation movement.

The GQDs in the coating act as solid lubricants when friction develops. While the grinding ball is moving, the GQDs reduce shear stress, provide lubrication at the contact surface, and protect the plating layer. Wear, the coefficient of friction, and the friction force on the plating layer may all be reduced more effectively by the uniformly distributed GQDs in the layer. When paired appropriately, Ni^2+^ and GQDs can significantly inhibit the occurrence of cracks. Furthermore, GQDs’ excellent ductility when extruded and distorted can result in elastic deformation, which can relieve deformation caused by external loads, protect the plating layer, and reduce the risk of fractures.

The XRD test findings indicated above show that the Ni-GQDs-III composite plating made by double-pulse electrodeposition contains tiny grains. The EDS test findings also indicate a significant carbon content in the plating. It demonstrates that there are more uniformly distributed solid lubrication sites throughout the plating, better-dispersed GQDs, and closely spaced grains throughout the plating. These properties can improve the plating’s resistance to wear while also effectively lowering the wear quantity and friction coefficient.

#### 3.5.4. Effect of Different Electrodeposition Processes on the Resistance to Corrosion of Plating

**Figure 12:**
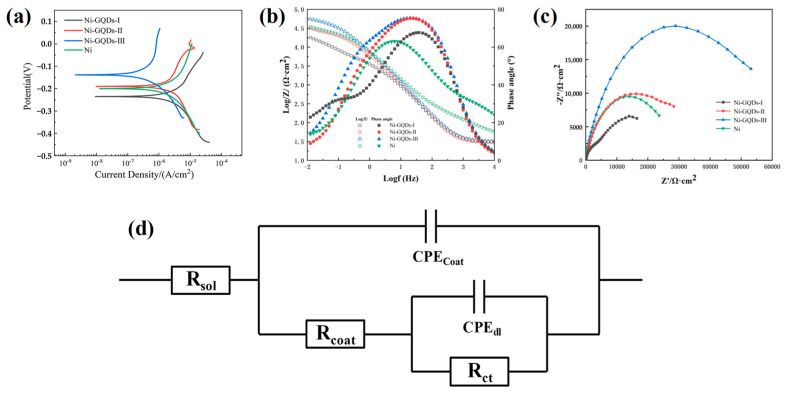
Plot of each plating’s electrochemical corrosion outcomes in a 3.5% NaCl solution; (**a**) Tafel polarization curve; (**b**) Bode Plots; (**c**) Nyquist diagrams; (**d**) equivalent electrochemical circuit.

The electrochemical corrosion outcomes of several plating layers in a 3.5% NaCl solution are displayed in Figure 12. The Tafel polarization curves for each plated layer are displayed in Figure 12a. These curves may be used to calculate the current densities and self-corrosion voltages of the plated layers created by the various electrodeposition methods [62]; the results are displayed in Table 9.

As noted in Figure 12a and Table 9, the Ni-GQDs-III composite plating generated by the double pulse electrolysis approach exhibited a higher self-corrosion voltage, −139 mV, than the plating produced by the other procedures. The Ni-GQDs-III composite plating’s self-corrosion voltages were 69.5% for Ni plating, 73.0% for Ni-GQDs-II composite plating, and 59.1% for Ni-GQDs-I composite plating, respectively. A notable 35 percent growth occurred. The stability of the plating surface is indicated by the self-corrosion stress; the higher the self-corrosion stress, the more stable and corrosion-resistant the plating [63]. In comparison to other plating, the Ni-GQDs-III composite plating shows greater corrosion resistance and a bigger self-corrosion stress. Concurrently, the self-corrosion current density of the Ni-GQDs-III composite plating decreases by 3.19 × 10^−7^ A/cm^2^. The Ni-GQDs composite plating’s self-corrosion current density was 20.9% of the Ni plating, 17.1% of the Ni-GQDs-I composite plating, and 25.6% of the Ni-GQDs-II composite plating. This indicates a significant decrease of 82.9% in the self-corrosion electricity intensity. Since the strength of the self-corrosion electricity is caused by material deterioration, the plating’s resistance to corrosion increases with resistance to charge transfer and decreases with self-corrosion electricity intensity [12]. The Ni-GQDs-III composite plating exhibits reduced self-corrosion current density and superior corrosion resistance in comparison to other plating layers.

In order to evaluate and research the corrosion resistance of the films created using different electrodeposition techniques, EIS studies were carried out on the plating. After that, the data were fitted and examined [64]. The Bode plots for the plating are shown in Figure 12b, the Nyquist plots for the plating generated by the various approaches are shown in Figure 12c, and the matching circuit diagrams utilized in the fitting are shown in Figure 12d. Based on the fitting findings, the values of the circuit element parameters in the pertinent matching circuits for the plating produced by the various methods are known; the specific values are shown in Table 9. The Ni-GQDs-III composite plating produced by the double-pulse electrolysis procedure clearly has a larger impedance radius than the plating produced by the other electrolysis processes, as shown in Table 9 and Figure 12c. This translates to a higher impedance value of 53,680 Ω·cm^2^. The impedance values are 227.3% for the Ni-GQDs-I composite plating, 219.1% for the Ni-GQDs-II composite plating, and 150.3% for the Ni plating, which is a significant improvement of 50.3%. Higher impedance plating has better resistance to corrosion because it can slow down the rate of oxidation and corrosion on the plating as well as the rate at which electrochemical processes occur on the outer layer of the plating by restricting the flow of electrons and ions and preventing the start of electrochemical processes [65].

**Figure 13:**
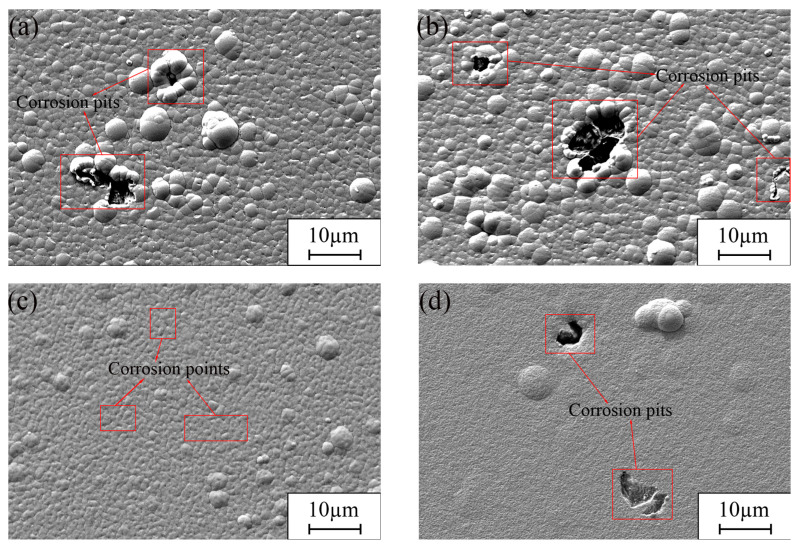
SEM pictures of various plating following a 120-h soak in a 3.5% NaCl solution; (**a**) Ni-GQDs-I; (**b**) Ni-GQDs-II; (**c**) Ni-GQDs-III; (**d**) Ni.

Figure 13 shows SEM images of the plated surfaces after 120 h of immersion corrosion in 3.5% NaCl solution. As shown in Figure 13a, two corrosion holes developed on the Ni-GQDs-I composite plating following the attack by corrosive solutions such as Cl^−^; one of the holes was larger and deeper than the other, and the plating’s surface structure was inadequate following corroding, suggesting that the plating’s resistance to corrosion was low [66]. After the immersion corrosion testing, the Ni-GQDs-II composite plating was seen to have two comparatively smaller and shallower corrosion pits on its surface in addition to one bigger and deeper corrosion pit (Figure 13b). The poor surface topography of the plating during corrosion also suggested that the plating’s corrosion resistance was inadequate. The Ni-GQDs-III composite plating after the immersion corrosion experiment is shown in Figure 13c, which makes it clear that the plating has good surface qualities after corrosion, such as fewer corrosion spots and no corrosion holes. This suggests that the plating has improved corrosion resistance. Following the immersion corrosion experiment, the Ni plating on its exterior developed two holes for corrosion, as shown in Figure 13d. The corrosion holes had similar widths, but one was deeper than the other, and the plating’s crystalline composition showed less improvement after corrosion, indicating that the Ni plating offered less protection against corrosion.

The Ni-GQDs-I composite plating produced by the DC plating method has defects such as holes and uneven bumps on the surface, as well as poor surface quality. Because the plating layer’s GQDs count is low, the nickel grain size distribution is uneven, the voids in the layer cannot be adequately filled, and the plating layer’s surface is unable to form a strong protective film to withstand the degradation of corrosive media, the corrosive effect of corrosive media, such as Cl^−^, is more pronounced and the plating layer’s corrosion resistance is insufficient [67]. The application of the single pulse electrodeposition method results in finely tuned plating grains, tight inter-grain connections, and minimal gaps at the grain boundaries. This configuration effectively prevents corrosive ions from penetrating the plating layer. Furthermore, the increased concentration of GQDs within the plating layer enhances its resistance to further corrosion and inhibits the infiltration of Cl^⁻^ ions.

The reverse pulse current can reduce concentration polarization and hydrogen precipitation, increase plating efficiency, remove protrusions and burrs from the plating layer’s surface, and produce a high-quality plating layer with a dense, flat surface free of visible defects when double-pulse electrodeposition is used to prepare composite plating layers. In addition, the plated layer has a larger GQDs content, microscopic grain size, high grain density, and a stronger surface protective layer against abrasive media. Double pulse electrodeposition, thus, results in better corrosion-resistant plating.

The absence of GQDs in the nickel plating produced by the double-pulse electrolysis technique resulted in an inability of the GQDs to effectively regulate the growth of nickel crystals, which increased the size of the nickel grains in the plating. Because GQDs cannot create a strong layer of protection on the surface or fill in the gaps in the plating, corrosive media like Cl^−^ can easily erode into the plating. As a result, the plating’s resistance to corrosion is diminished.

**Figure 14:**
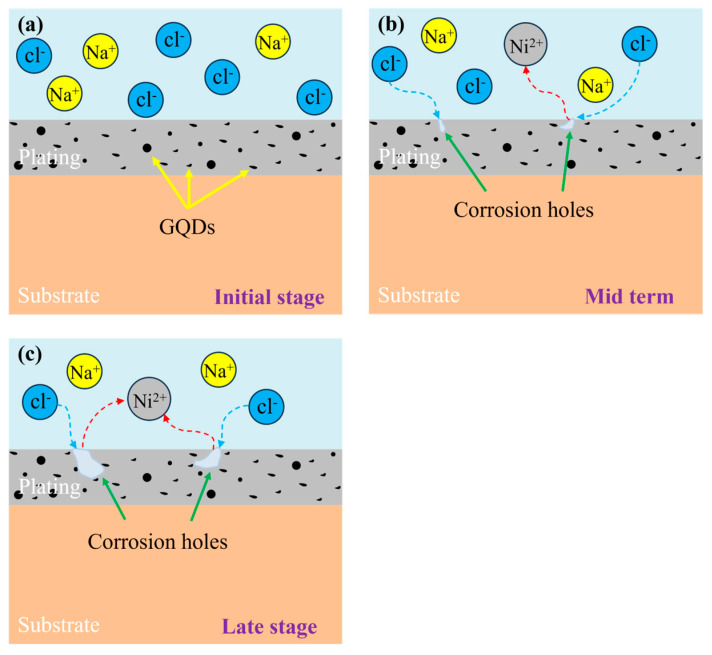
Diagram of the electrochemical corrosion mechanism for Ni-GQDs composite plating. (**a**) Initial stage, (**b**) Mid-term, (**c**) late stage of corrosion.

The electrochemical corrosion mechanism diagram for the Ni-GQDs composite plating is shown in Figure 14. Due to the strong corrosiveness of Cl^−^ in NaCl solution, Cl^−^ continually permeated the plating layer during the corrosion resistance test tests and partially damaged it. At the start of the test, the plating’s outer surface contained Na^2+^ and Cl^−^ from the NaCl solution, which was in contact with it and actively corroding. Subsequently, Cl^−^ started to seek for the plating layer’s weak spot and started to erode, small corrosion pits started to form on the layer’s surface, and metallic nickel oxidized to Ni^2+^, which was combined with the corrosion solution. After corrosion for longer than a certain amount of time, the degree of corrosion of the plating layer progressively grows, the level of Ni^2+^ in the solution rises, and the plating layer is clearly damaged as the contact surface between corrosion pits and Cl^−^ increases.

The corrosion of the plating layer produced by Cl^−^ can be tolerated by plating layers that have tiny grains, tight connections between grains, and a large number of grains per unit area. The plating layer’s resistance to corrosion may be improved, and the degree of corrosion can be decreased by the presence of GQDs. They also effectively reduce the interaction between corrosive ions and the plating layer. Superior GQDs and the nickel matrix work together to shield the plating layer from corrosive ions and stop Cl^−^ from corroding it further when corrosion occurs.

From the aforementioned, it is clear that Ni-GQDs-III has smaller grains, that the combination of nickel grains and GQDs is superior, and that there are only little corrosion holes on the plating layer’s surface that do not continue to grow. This indicates how well Ni-GQDs composite plating created with double pulse electrodeposition resists corrosion.

## 4. Conclusions

The supercritical CO_2_ atmosphere utilized in the creation of Ni-GQDs composite plating significantly enhances the mechanical properties, microstructural morphology, and corrosion resistance of the plating. Although the direct current (DC) method for manufacturing the Ni-GQDs-I composite plating is efficient and cost-effective, it results in suboptimal surface morphology and mechanical properties. The Ni-GQDs-II composite plating made by this method has a better surface shape and better features because the instantaneous high-current characteristic of the single-pulse power supply helps to refine the grain size of the plating and deposit more GQDs. The double-pulse electrodeposition process effectively removes pits and other defects on the plated surface, refines the grain size of the plated layer, and deposits more GQDs so that the Ni-GQDs-III composite plating has a smooth appearance with no obvious defects and has better mechanical properties and corrosion resistance. In double-pulse current electroplating of pure nickel, where the plating solution lacks GQDs, current efficiency is directly reflected in grain growth, leading to increased grain size and a corresponding decline in microhardness, abrasion resistance, and corrosion resistance of the plated layer. The results demonstrate the superior performance of Ni-GQDs composite plating produced under supercritical CO_2_ conditions via double-pulse electrodeposition. The surface of the composite plating revealed a flat, densely organized structure, along with a reduction in defect count and an improvement in the quality of GQDs. Notably, the granules of the composite plating were smaller than those of pure nickel plating. The hardness of the composite plating was measured at 867.2 HV, representing an 11.4% enhancement over the hardness of pure nickel plating. Additionally, the volumetric wear and coefficient of friction were recorded at 3.395 × 10^7^ µm^3^ and 0.26, respectively, reflecting significant reductions of 27.6% and 41% compared to the values observed for pure nickel plating. Furthermore, the composite plating exhibited a higher self-corrosion potential of −139 mV, indicating a noteworthy increase of 30.5%. The self-corrosion current density decreased significantly by 82.9%, reaching 3.19 × 10⁻^7^ A/cm^2^.

## Figures and Tables

**Table 1:** Chemical Reagent Formulations for GQDs Solutions.

Chemical Reagent	Dosage (g·L^−1^)	Weight (g)
C_6_H_8_O_7_·H_2_O	42	4.2
CH_4_N_2_O	36	3.6

**Table 2:** Formulations for supercritical electrodeposition solutions.

Chemical Reagent	Dosage (g/L)	Content (g)
NiSO_4_·6H_2_O	300	45
NiCl_2_·6H_2_O	30	4.5
H_3_BO_3_	35	5.25
C_12_H_25_SO_4_Na	0.20	0.03
GQDs	1.50	0.225

**Table 3:** Electroplating parameters of Ni-GQDs composite plating and Ni plating.

Samples	Ni-GQDs-I	Ni-GQDs-II	Ni-GQDs-III	Ni
Forward pulse current density/(A/dm^2^)	5	5	5	5
Forward pulse duty cycle	/	0.25	0.25	0.25
Reverse pulse current density/(A/dm^2^)	/	/	0.8	0.8
Reverse pulse duty cycle	/	/	0.25	0.25
Forward pulse frequency/kHz	/	1	1	1
Reverse pulse frequency/kHz	/	/	1	1
GQDs (g/L)	1.5	1.5	1.5	0
Stresses/MPa	10.5	10.5	10.5	10.5
Temperature/°C	50	50	50	50
Time/min	60	60	60	60

**Table 4:** Percentage of elements C and Ni.

Elements	at%	wt%
C	10.50	2.34
Ni	89.50	97.66
Overall amount	100	100

**Table 5:** Grain sizes and RTC of Ni plating and Ni-GQDs composite plating.

Samples	hlk	2θ (°)	FWHM	Crystallite Size (nm)	Average Grain Size (nm)	I_s_ (hkl)	I_0_ (hkl)	RTC/%
	111	44.86	1.019	8.34		4214.29	100	57.27
Ni-GQDs-I	200	52.13	2.249	3.89	5.37	785.01	42.3	25.22
	220	76.64	2.573	3.89		208.69	16.2	17.51
	111	44.85	1.042	8.15		4101.92	100	58.88
Ni-GQDs-II	200	52.07	2.283	3.83	5.27	697.22	42.3	23.66
	220	76.68	2.625	3.82		197.01	16.2	17.46
	111	44.77	1.095	7.76		5733.78	100	64.36
Ni-GQDs-III	200	51.88	2.594	3.37	4.58	848.82	42.3	22.52
	220	76.55	3.821	2.62		189.28	16.2	13.12
	111	45.14	0.964	8.83		2080.51	100	15.75
Ni	200	52.31	0.992	8.83	6.88	4339.19	42.3	77.68
	220	76.35	3.352	2.98		140.52	16.2	6.57

**Table 6:** The position and intensity values of peak D and peak G in the Raman spectrogram.

Samples	D-Band Position (cm^−1^)	G-Band Position (cm^−1^)	D-Band Strength (I_D_)	G-Band Strength (I_G_)	I_D_/I_G_
GQDs	1336.64	1553.88	4510.96	5471.48	0.8244
Ni-GQDs-I	1299.54	1587.01	2611.88	3197.28	0.8169
Ni-GQDs-II	1300.92	1589.66	1704.18	2362.74	0.7213
Ni-GQDs-III	1327.04	1573.78	2762.10	4224.80	0.6538

**Table 7:** The Ra and Rq of the different plating.

Sample Numbers	Ra, nm	Rq, nm
Ni-GQDs-I	92.7	116
Ni-GQDs-II	80.3	101
Ni-GQDs-III	47.3	63.8
Ni	103	124

**Table 8:** Maximum wear mark depth, wear mark cross-sectional area, and wear volume of plating made using various electrodeposition methods.

Samples	Maximum Abrasion Mark Depth (µm)	The Abrasion Marks’ Cross-Sectional Area (µm^2^)	Volumetric Wear of Plating (µm^3^)
Ni-GQDs-I	20	5288	5.288 × 10^7^
Ni-GQDs-II	19.3	5069	5.069 × 10^7^
Ni-GQDs-III	16.0	3395	3.395 × 10^7^
Ni	18.8	4687	4.687 × 10^7^

**Table 9:** Corrosion parameters for plating layers at different electrodeposition methods.

Samples	*E_corr_*, mV	*I_corr_*, 10^−7^ A/cm^2^	*R_Sol_*, Ω·cm^2^	*CPE_coat_*, 10^−5^Ω^−1^ cm^−2^S^−n^	*n_coat_*	*R_coat_*, Ω·cm^2^	*CPE_dl_*, 10^−5^Ω^−1^ cm^−2^S^−n^	*n_dl_*	*R_ct_*, 10^4^Ω·cm^2^	*e*, %	*Chisq*, ×10^−4^
Ni-GQDs-I	−235	18.7	30.41	2.79	0.8621	3832	17.39	0.6054	2.362	<1.727	2.98
Ni-GQDs-II	−189	12.5	29.76	2.087	0.9137	6729	3.425	0.6112	2.45	<2.605	6.79
Ni-GQDs-III	−139	3.19	31.67	1.825	0.9057	8407	1.948	0.6135	5.368	<0.893	0.80
Ni	−200	15.2	38.34	2.652	0.6564	330.7	0.692	0.8928	3.572	<4.982	24.82

## Data Availability

Data are contained within the article.

## Supplementary Materials

The following supporting information can be downloaded at: https://www.mdpi.com/article/10.3390/ma17184620/s1, Figure S1: Physical and TEM images of GQDs. Figure S2: Content of graphene quantum dots (GQDs) in coatings prepared by different processes. Figure S3: Distribution map of C and Ni elements in Ni GQDs-III nanocomposite coating. Figure S4: EDS planar scanning results of Ni-GQDs-III nanocomposite coating. Figure S5: Raman spectroscopy results of graphene quantum dots (GQDs) powder and Ni-GQDs nanocomposite coatings prepared by different electrodeposition processes. Figure S6: AFM images of coatings prepared by different electrodeposition processes.
