# Supplementary material for: Effect of Process Conditions on the Microstructure and Properties of Supercritical Ni-GQDs Plating
Source: Materials (Basel). 2024 Sep 20;17(18):4620. doi: 10.3390/ma17184620 (PMC11433132; doi:10.3390/ma17184620)
Supplement: Supplementary file 1 [file materials-17-04620-s001.zip › materials-3164401-supplementary.pdf]

Effect of Process Conditions on the Microstructure and Properties of Supercritical Ni-GQDs Plating

Haoyu Zhong [1], Cong Fang [1], Weining Lei [1,2,*], Tianle Xv [1], Bin He [1,2], Linglei Kong [1,2] and Yiliang He [1]

[1] School of Mechanical Engineering, Jiangsu University of Technology, Changzhou 213001, China; hy_zhong_personal@163.com (H.Z.); 18263851593@163.com (C.F.); 18339188030@163.com (T.X.); binhe06@163.com (B.H.); konglingl@nuaa.edu.cn (L.K.); 13485987469@163.com (Y.H.)

[2] Jiangsu Key Laboratory of Advanced Material Design and Additive Manufacturing, Jiangsu University of Technology, Changzhou 213001, China

* Correspondence: leiweining@jsut.edu.cn

Supporting Information

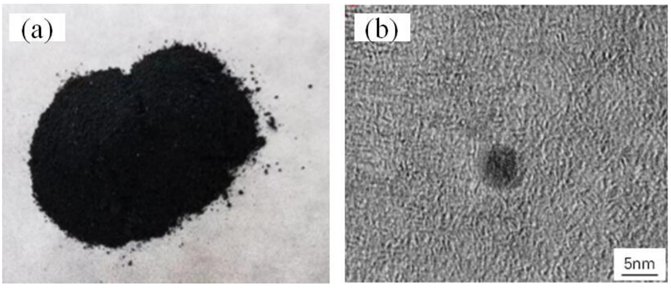

Figure. S1 Physical and TEM images of GQDs.

(a) Physical image; (b) TEM image.

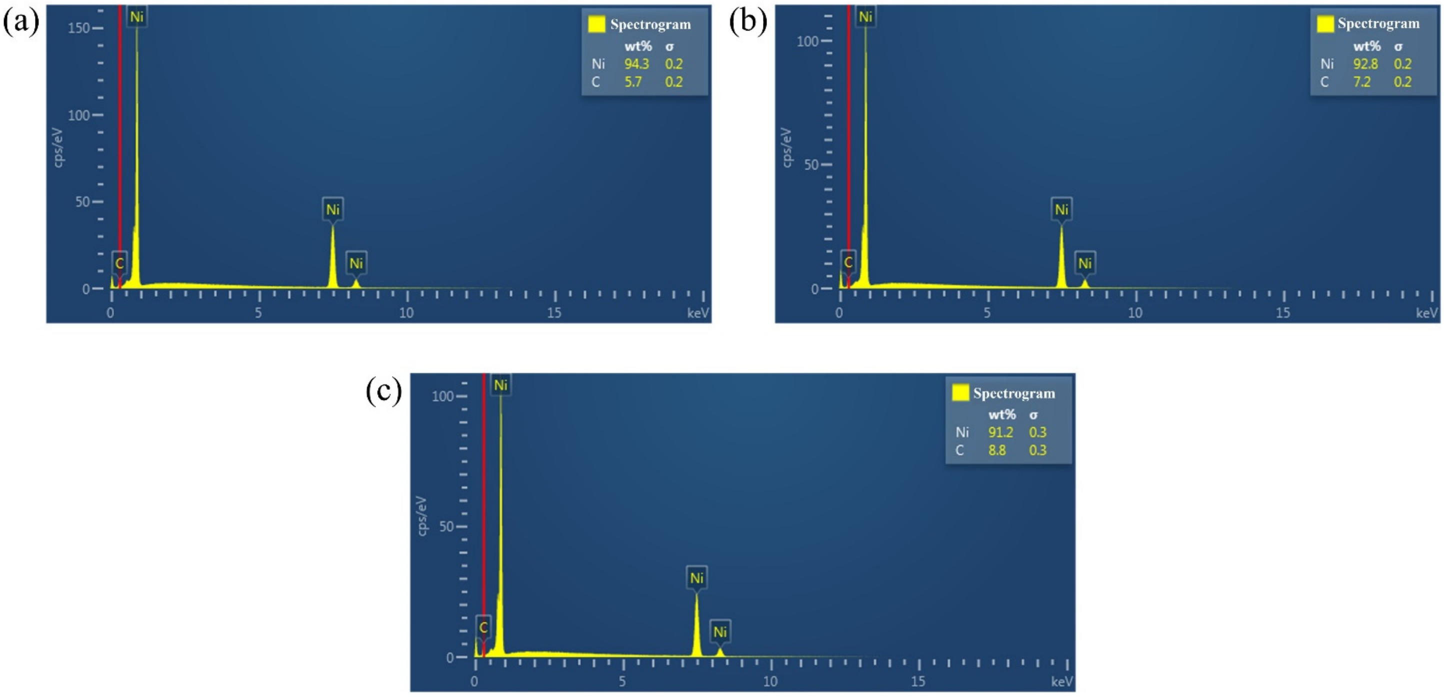

Figure. S2 Content of graphene quantum dots (GQDs) in coatings prepared by different processes;

(a)Ni-GQDs-I; (b) Ni-GQDs-II; (c) Ni-GQDs-III.

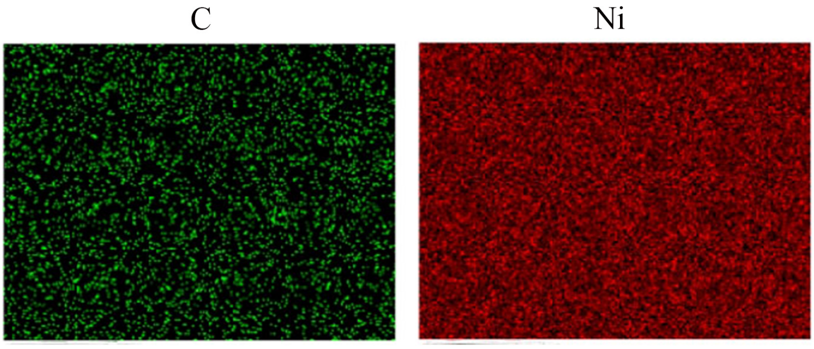

Figure. S3 Distribution map of C and Ni elements in Ni GQDs-III nanocomposite coating.

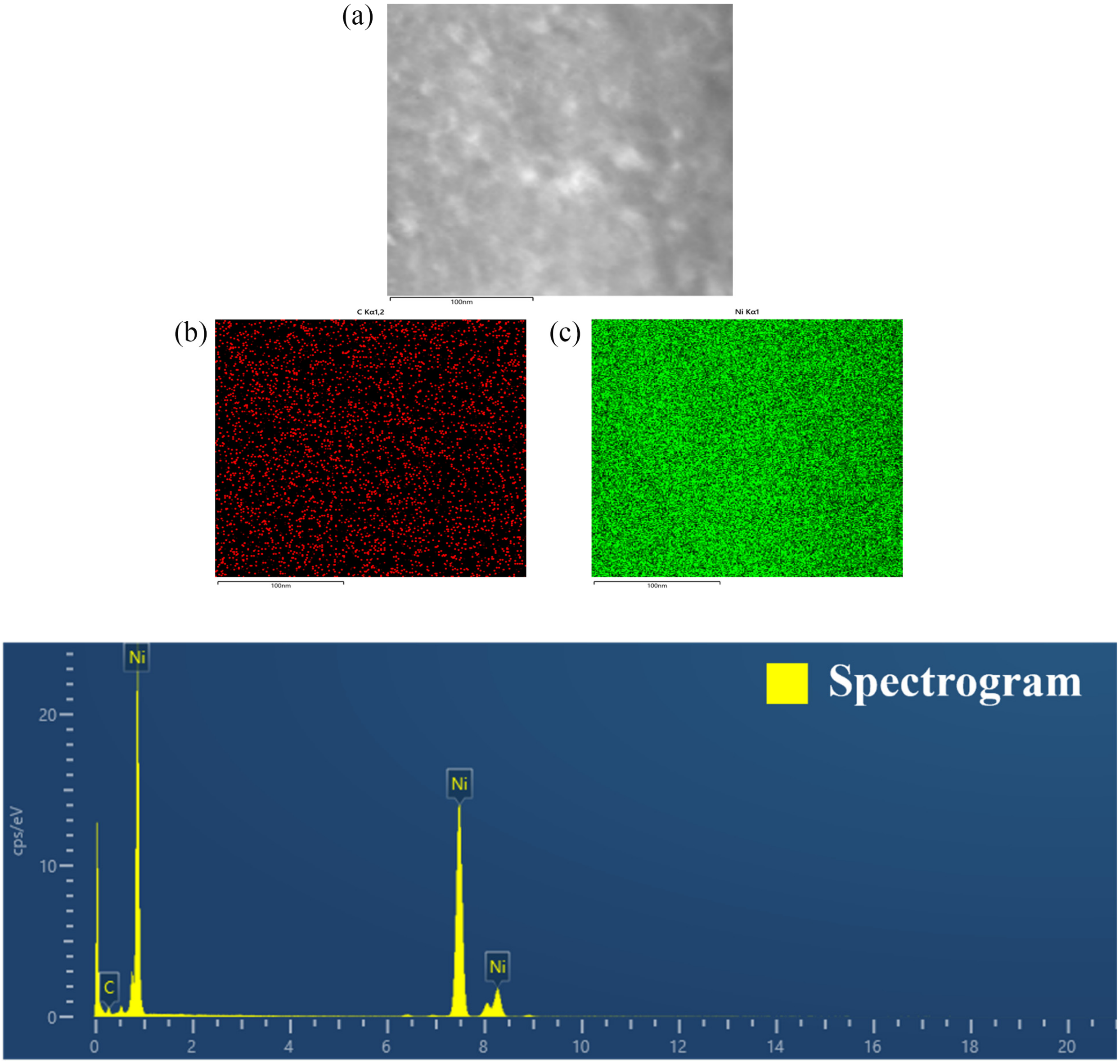

Figure. S4 EDS planar scanning results of Ni-GQDs-III nanocomposite coating; (a) Surface morphology of the coating; (b) Distribution of element C in the coating; (c) Distribution of element Ni in the coating; (d) Percentage of element C and Ni.

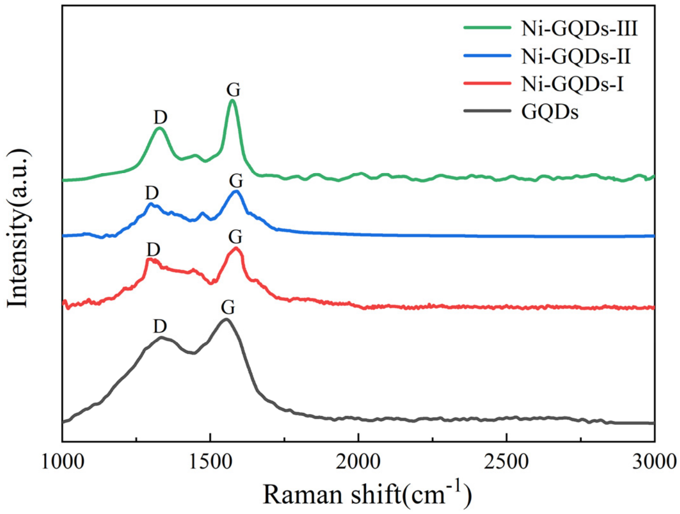

Figure. S5 Raman spectroscopy results of graphene quantum dots (GQDs) powder and Ni-GQDs nanocomposite coatings prepared by different electrodeposition processes.

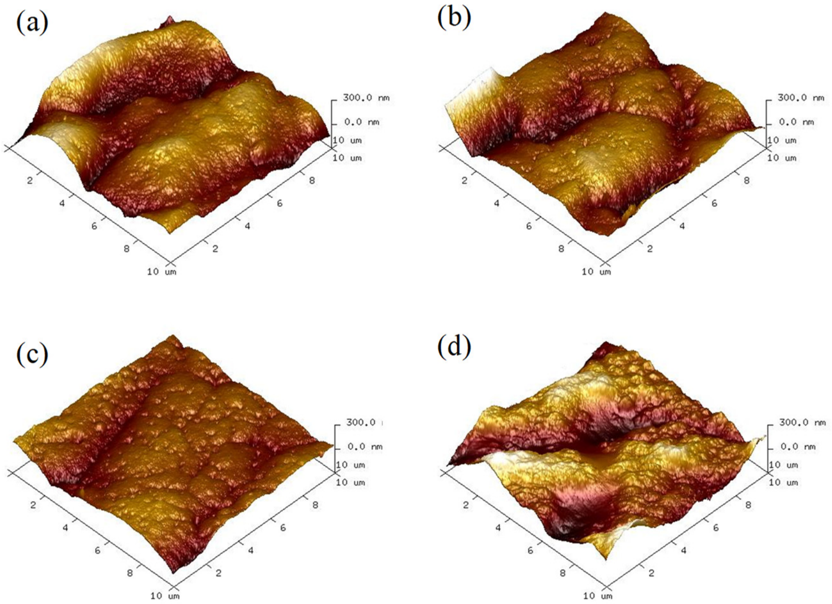

Figure. S6 AFM images of coatings prepared by different electrodeposition processes.
